# Complete Genome Sequence of Staphylococcus aureus Siphophage Sebago

**DOI:** 10.1128/MRA.00765-19

**Published:** 2019-07-18

**Authors:** Kathryn Klotz, Abby Korn, Heather Newkirk, Mei Liu, Jason J. Gill, Jolene Ramsey

**Affiliations:** a Center for Phage Technology, Texas A&M University, College Station, Texas, USA; Queens College

## Abstract

Here, we introduce the genome of Sebago, a 43,878-bp siphophage that infects Staphylococcus aureus. Sebago carries 70 proteins and is most closely related to StauST398, a *Phietavirus*.

## ANNOUNCEMENT

Staphylococcus aureus is a Gram-positive, nonsporulating, commensal microbe that affects both humans and animals (1). While typically present in the normal flora of human skin and nares, various strains of S. aureus, such as methicillin-resistant S. aureus (MRSA), have obtained antibiotic resistance and become leading causes of nosocomial infections (2). To combat the antibiotic resistance in S. aureus, alternative treatments using bacteriophage are in development (3). Here we present the complete genome of Sebago, a siphophage infecting S. aureus.

Sebago was isolated from a filtered (0.2-μm) Minnesota swine barn environmental swab eluate via an overnight enrichment against S. aureus strain PD17 (Texas swine nasal isolate; *spa* type t034) (4). The phage and host propagation were carried out aerobically at 30°C in tryptic soy broth (TSB) medium (Difco) using the soft-agar overlay method (5). The genomic DNA for Sebago was purified with the Promega Wizard DNA cleanup kit according to the modification in the shotgun library preparation protocol given by Summer (6), with an additional 10 mM EDTA (pH 8.0) and 100 μg/ml proteinase K treatment for 30 min at 50°C after polyethylene glycol (PEG) precipitation (to eliminate heat-stable staphylococcal nucleases). To generate 250-bp paired libraries, we used an Illumina TruSeq Nano low-throughput kit (6). The Illumina MiSeq index using v2 500-cycle chemistry yielded 372,373 total reads. FastQC (http://www.bioinformatics.babraham.ac.uk/projects/fastqc/) was used for quality control. FastX-Toolkit v0.0.14 (http://hannonlab.cshl.edu/fastx_toolkit/) was used for trimming. The Sebago contig was assembled using SPAdes v3.5.0 with 87-fold coverage (7). The genome was confirmed as complete via PCR (forward primer, 5′-CTGCCAAAGTCTGTAGCAATAAC-3′; reverse primer, 5′-TTGCTTACTGGCGACTTCTC-3′) and Sanger sequencing of the product. Annotation was done in Web Apollo, first calling genes with GLIMMER v3.0, MetaGeneAnnotator v1.0, and ARAGORN v2.36 for tRNAs (8–11). Terminators predicted as rho-independent were identified with TransTermHP (12). Functional prediction made use of evidence from TMHMM v2.0, InterProScan v5.22, and BLAST against the NCBI nonredundant and UniProtKB Swiss-Prot/TrEMBL databases (13–16). The annotation tools were run in the Galaxy instance hosted using default parameters by the Center for Phage Technology at Texas A&M University (https://cpt.tamu.edu/galaxy-pub/) (17). To ascertain the phage morphology, Sebago samples were negatively stained with 2% (wt/vol) uranyl acetate and viewed by transmission electron microscopy at the Texas A&M Microscopy and Imaging Center (18).

Sebago has a 43,878-bp genome with a 94.5% coding density. There are 70 protein-coding genes, with 37 functional predictions made, and no tRNA genes. Sebago’s 35.3% G+C content is similar to the ∼33% of its host S. aureus (19). PhageTerm predicts a headful packaging mechanism for this phage (20). In progressiveMauve and BLASTp comparisons, Sebago has 77.4% overall nucleotide identity and 54 genes similar to those of another S. aureus siphophage, StauST398 (GenBank accession no. JQ973847) (21, 22). StauST398, a *Phietavirus*, has a genome size, total number of genes, and G+C content similar to those of Sebago.

Interestingly, in the Sebago genome, the putative tape measure protein (GenBank accession no. QBQ72253) is adjacent to the predicted tail assembly chaperone (QBQ72255) and its frameshifted product (QBQ72254), similar to the phage lambda G/GT chaperone system (23).

### Data availability.

The genome sequence and associated data for phage Sebago were deposited under GenBank accession no. MK618716, BioProject accession no. PRJNA222858, SRA accession no. SRR8869228, and BioSample accession no. SAMN11360406.
